# Immune involvement in the pathogenesis of schizophrenia: a meta-analysis on postmortem brain studies

**DOI:** 10.1038/tp.2017.4

**Published:** 2017-03-28

**Authors:** C F M G van Kesteren, H Gremmels, L D de Witte, E M Hol, A R Van Gool, P G Falkai, R S Kahn, I E C Sommer

**Affiliations:** 1 Department of Psychiatry, Brain Center Rudolf Magnus Institute, University Medical Center Utrecht, Utrecht, The Netherlands; 2 Department of Nephrology and Hypertension, University Medical Center Utrecht, Utrecht, The Netherlands; 3 Department of Translational Neuroscience, Brain Center Rudolf Magnus, University Medical Center Utrecht, Utrecht, The Netherlands; 4 Department of Neuroscience, Netherlands Institute for Neuroscience, an Institute of the Royal Netherlands Academy of Arts and Sciences, Amsterdam, The Netherlands; 5 Faculty of Science, Swammerdam Institute for Life Sciences, Center for Neuroscience, University of Amsterdam, Amsterdam, The Netherlands; 6 Department of Psychiatry, Yulius Mental Health Organization, Barendrecht, The Netherlands; 7 Department of Psychiatry and Psychotherapy, Ludwig Maximilian University, Munich, Germany

## Abstract

Although the precise pathogenesis of schizophrenia is unknown, genetic, biomarker and imaging studies suggest involvement of the immune system. In this study, we performed a systematic review and meta-analysis of studies investigating factors related to the immune system in postmortem brains of schizophrenia patients and healthy controls. Forty-one studies were included, reporting on 783 patients and 762 controls. We divided these studies into those investigating histological alterations of cellular composition and those assessing molecular parameters; meta-analyses were performed on both categories. Our pooled estimate on cellular level showed a significant increase in the density of microglia (*P*=0.0028) in the brains of schizophrenia patients compared with controls, albeit with substantial heterogeneity between studies. Meta-regression on brain regions demonstrated this increase was most consistently observed in the temporal cortex. Densities of macroglia (astrocytes and oligodendrocytes) did not differ significantly between schizophrenia patients and healthy controls. The results of postmortem histology are paralleled on the molecular level, where we observed an overall increase in expression of proinflammatory genes on transcript and protein level (*P*=0.0052) in patients, while anti-inflammatory gene expression levels were not different between schizophrenia and controls. The results of this meta-analysis strengthen the hypothesis that components of the immune system are involved in the pathogenesis of schizophrenia.

## Introduction

Schizophrenia is a severe psychiatric disorder with a worldwide prevalence just below 1%, that often leads to dysfunction and suffering for patients and their families and places a significant burden on global health.^1^ Although the introduction of antipsychotic medications in the 1950s has substantially improved the treatment of positive symptoms of schizophrenia,^2^ the disease still causes considerable morbidity and mortality.^3^ Given this extensive impact, the need for better treatment for patients with schizophrenia is high.

The pathogenesis of schizophrenia is only partly understood: animal models can so far only be used to study certain aspects of the disease, and interpretations about the neurobiological basis of schizophrenia derived from these models should therefore be made with caution.^4^ Neuropathological research in postmortem material of schizophrenia patients is therefore of importance, as it could bridge the gap between molecular abnormalities in brain tissue and clinical symptomatology of psychotic disorders.^5^

One of the suggested underlying disease mechanisms in schizophrenia is a deregulation of immune processes in the central nervous system (CNS). Different lines of evidence support this hypothesis. Genetic studies consistently observe the strongest association with schizophrenia in the major histocompatibility complex (MHC) region on chromosome 6p21.3-22.1,^6, 7^ which was recently linked to the complement system, in particular to complement factor 4.^8^ Additional suggestion of immune involvement comes from nation-wide cohort studies reporting an increased risk of schizophrenia patients and their relatives for autoimmune diseases and vice versa.^9^ We have long known that an infectious trigger early in life has been associated with increased vulnerability to schizophrenia given the consistent association between schizophrenia and pre- and perinatal infections.^10^ Moreover, there is an increased frequency of seroconversion, that is, the time period during which a specific antibody develops and becomes detectable in blood, to certain pathogens in patients with schizophrenia.^11^

The immune system of the CNS is complex and is at present only partly elucidated. Key players are resident microglia, and perivascular and invading macrophages. Although these cells originate from different cellular lineages, they have a role in both innate and adaptive immunity, depending on their state of activity. Microglia are myeloid cells that have entered the CNS during early embryonic development. In response to dangerous stimuli such as infection, acute trauma or neurodegenerative processes, microglia migrate to the site of injury and often proliferate. During states of acute inflammation, other myeloid cell numbers may also further increase as a result of the influx of monocytes into the CNS that differentiate into macrophages. The presence of microglia can be visualized, using positron emission tomography (PET). Increased microglial density in patients with schizophrenia is found by different PET studies.^12, 13, 14^ Reports in literature are conflicting, however, depending on the tracer used and which phase of the disease is studied. PET studies have limiting factors such as small sample size, non-specific tracer binding and variability in binding affinity patterns between humans. Yet, these findings can be of relevance, as they may point out signs of altered activity of the immune system both prodromal and throughout disease.

In addition to these myeloid cell types, other resident cells of the CNS (microglia, for example, astrocytes and oligodendrocytes) also exert immunological functions. Reactive astrogliosis, characterized by astrocyte hypertrophy often coincides with this process. The mere activation of glial cells and the presence of higher numbers of myeloid cells can affect neuronal communication and regeneration of the brain.^15, 16^ It is becoming increasingly clear that immune processes in the brain are not only involved in inflammatory responses, but also in tissue repair, homeostasis, neuroplasticity, synaptic pruning and other neurodevelopmental processes.^12, 17^ MHC class I molecules and complement, for example, regulate many aspects of brain development, including neurite outgrowth, synapse formation and function, homeostatic plasticity, and activity-dependent synaptic refinement.^8, 17, 18, 19^

Neuropathology research by means of postmortem brain examination can be an important tool to examine alterations in immune cells and immunological pathways. Human postmortem brain material of schizophrenia patients is relatively sparse as compared with other brain diseases such as Alzheimer's. When reviewing the literature, several postmortem studies assessed alterations of immune processes in brain tissue of patients with schizophrenia, yet with inconsistent results. In this manuscript, we perform a quantitative review in a meta-analysis to provide a broad overview of findings regarding the involvement of different components of the immune system in the brains of schizophrenia patients and compare them with healthy controls.

## Materials and methods

### Literature search

This quantitative review was performed according to the Preferred Reporting Items for Systematic Reviews and Meta-Analyses (PRISMA) Statement.^20^ A literature search was conducted using PubMed, Embase and National Institutes of Health ClinicalTrials.gov. No year or language restrictions were applied. Duplicate studies were removed manually. The following keywords were used in the search, both alone and in combinations:

‘Schizophrenia', ‘psychosis', ‘autopsy', ‘postmortem', immune system', ‘inflammation', ‘cytokines', ‘interleukins', ‘chemokines', ‘complement', ‘microglia', ‘gliosis', ‘astrocytes', ‘macrophages', ‘lymphocytes', ‘leukocytes', ‘MHC I', ‘MHC II' and ‘HLA'.

In addition, the reference lists of identified papers, reviews and meta-analyses were screened for cross-references. When necessary, authors were contacted to provide additional details. The search was updated until 1 January 2016.

### Inclusion

Candidate studies had to meet the following inclusion criteria:

1. Studies performed on human postmortem brain material, with a healthy control group matched for age and sex.
2. Included patients had a diagnosis of a schizophrenia spectrum disorder (schizophrenia, schizoaffective disorder or schizophreniform disorder) either by clinical diagnosis, retrospective chart review, according to the diagnostic criteria of the Diagnostic and Statistical Manual of Mental Disorders (DSM-III, DSM-III-R, DSM-IV, DSM-IV-TR; American Psychiatric Association, 1994), or the International Classification of Diseases (ICD-9 or ICD-10; World Health Organization, 1992).
3. Sufficient information was reported in the article or by the authors upon request to compute common effect size statistics, that is, means and s.d., exact *P*-, *t*- or *z*-values conform Lipsey and Wilson.^21^ Whole-genome microarray and RNAseq data sets were not taken into account as we are unable to account for the multiple testing problem arising in explorative, non-hypothesis-directed research in a quantitative way.

### Data extraction

Raw data were extracted from tables or article text and converted to standardized mean differences (SMDs), computed as 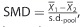, where 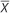 is the sample mean of a given group and s.d._pool_ is the within-group s.d., pooled across groups. The latter is derived by 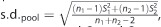, where *n*_1_ and *n*_2_ are the sample sizes in the two groups and *S*_1_ and *S*_2_ are the two s.d. Where provided, standard errors (s.e.) were converted to s.d. by 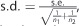, where *n*_1_ and *n*_2_ are the number of subjects in control and schizophrenia groups respectively. If no summary data for groups were presented, the exact *P*-value was converted to a test statistic by taking the score from a *t*-distribution with n_1_+n_2_−2 degrees of freedom. Missing data were requested from the authors, or approximated from graphs using GraphClick 3.0 (Arizona Software, Zürich, Switzerland).

### Subtyping of assessments in included studies

Study characteristics were grouped into larger umbrella categories,^22^ for example, those assessing cells and those assessing molecular targets. Molecular targets were subdivided into those studying protein- or RNA expression. One study assessed a hormone. In addition due to the large heterogeneity of markers in the molecular sample, every measured parameter was classified as ‘proinflammatory', ‘anti-inflammatory' or ‘neutral', based on previous associations in literature. For cellular targets the subdivision was performed based on cell types, namely microglia or macroglia, the latter comprising glial cells such as astrocytes and oligodendrocytes but also studies that assessed ‘glial cells' or ‘gliosis', which was not further specified. One study assessed lymphocytes. In addition the brain areas under investigation in different studies were grouped into larger categories reflecting major brain structures.^22^

### Data analysis

Data were analyzed with ‘R' software (version 3.1.0, R Foundation for Statistical Computing, Vienna, Austria), using the metafor package.^23^ Random- and mixed effects models were fitted using restricted maximum likelihood estimation (REML). As many studies included multiple similar outcome measures (for example, immunohistochemical stainings for the same cell type) independence of the data was not given. We therefore accounted for within-study clustering by creating a multilevel model^24, 25^ that nested related outcome measures within the same cohort of studies.

Data are presented as SMD+95% confidence interval (CI). Estimated average effect (*μ*), heterogeneity in effects (*τ*^2^) and the estimated percentage of variability attributable to heterogeneity (*I*^2^) are given. Heterogeneity was considered to be significant if *I*^2^>50%.^26^ Subgroup- and sensitivity analysis were conducted to investigate potential sources of heterogeneity. The effects of the following moderators were studied using meta-regression: brain area, pH, duration of illness, gender, suicide and age at death.

In order to screen for irregularities in inter-study effects, a contour-enhanced funnel-plot-based approach was used. Egger's test was used as a simple test of funnel plot asymmetry^27^ and the trim-and-fill method by Duval and Tweedie^28^ was used to impute ‘missing' studies in the case of pronounced funnel-plot asymmetry. Sensitivity analysis to outliers and influential cases was performed by calculating Cook's distances for individual studies.^23^

## Results

**Figure 1:**
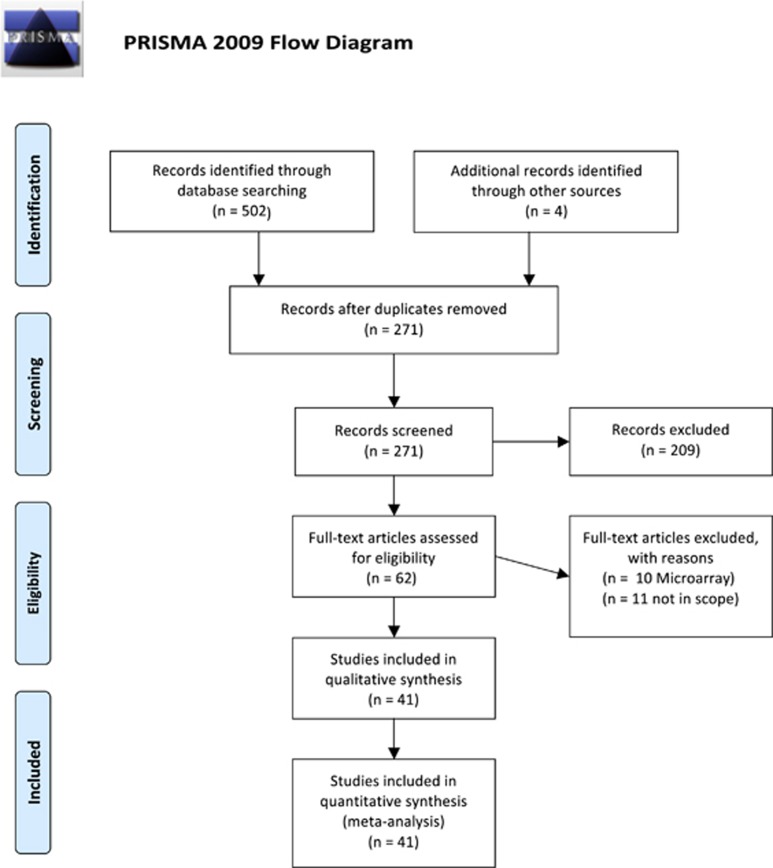
Flow-chart of study-selection.

Our search strategy resulted in the identification of 268 unique study abstracts, 231 of which were excluded, as they did not meet our inclusion criteria. We performed a meta-analysis on 41 studies, including a total of 783 patients and 762 controls (Figure 1). We divided the studied parameters into two categories; cellular/histological and molecular components in order to provide a comprehensible overview.

### Cellular parameters

**Figure 2:**
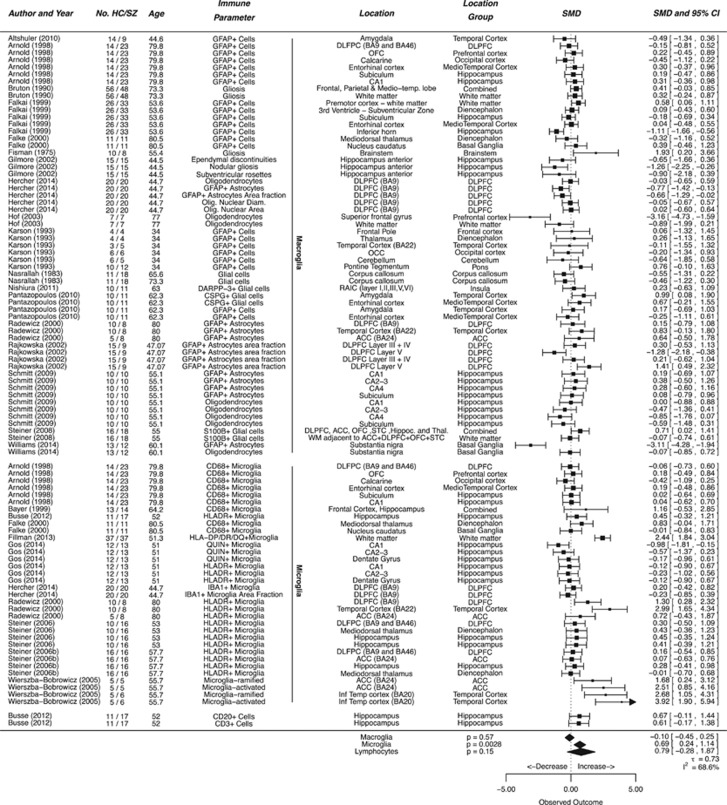
Cellular parameters in postmortem brains of schizophrenia patients. Forest plot showing cellular parameters measured in the brains of schizophrenic patients compared to controls. Values are expressed as standardized mean differences (SMDs) with 95% confidence intervals (CI). The column ‘No. HC/SZ' denotes the number of patients in the healthy control and schizophrenic groups. The column ‘Immune Parameter' denotes the staining or scoring method used to quantify immune involvement. The plot is divided in subsections showing study cohorts in which microglia, macroglia and lymphocytes are measured. The column ‘Location' indicates the brain area under investigation as reported by the authors of the individual study, ‘Location Group' indicates the re-classification assigned by us for meta-regression. The diamonds at the bottom indicate the pooled estimate obtained by multilevel random effects meta-analysis.

Twenty five studies investigated alterations in cell density, including a total of 402 patients and 382 controls. In order to generate meaningful summary estimates we further subdivided the three cell types into ‘Microglia', ‘Macroglia' consisting of other glial cells such as astrocytes and oligodendrocytes, and ‘Lymphocytes' (Figure 2).

The subcategory of ‘Microglia' included 11 studies on 181 patients and 159 controls. A significant, moderate to large increase in microglia was observed (effect size=0.69 SMDs (95% CI 0.24–1.14), *P*=0.0028) over all studies (Figure 2). There was significant heterogeneity with *I*^2^ 68.6 %.

The subcategory of ‘Macroglia' included 18 studies on 283 patients and 278 controls. Findings did not differ significantly (effect size=−0.10 SMDs (95% CI −0.45 to 0.25), *P*=0.57) between schizophrenia patients and controls.

The category of lymphoid cells contained only one study,^29^ in which both CD3^+^ T-cells as well and CD20^+^ B-cells were measured, which accounted for an SMD 0.61 (−0.17 to 1.38) CD3^+^ T-cells, respectively, SMD 0.67 (−0.11 to 1.44) for CD20^+^ B-cells.

### Molecular parameters

**Figure 3:**
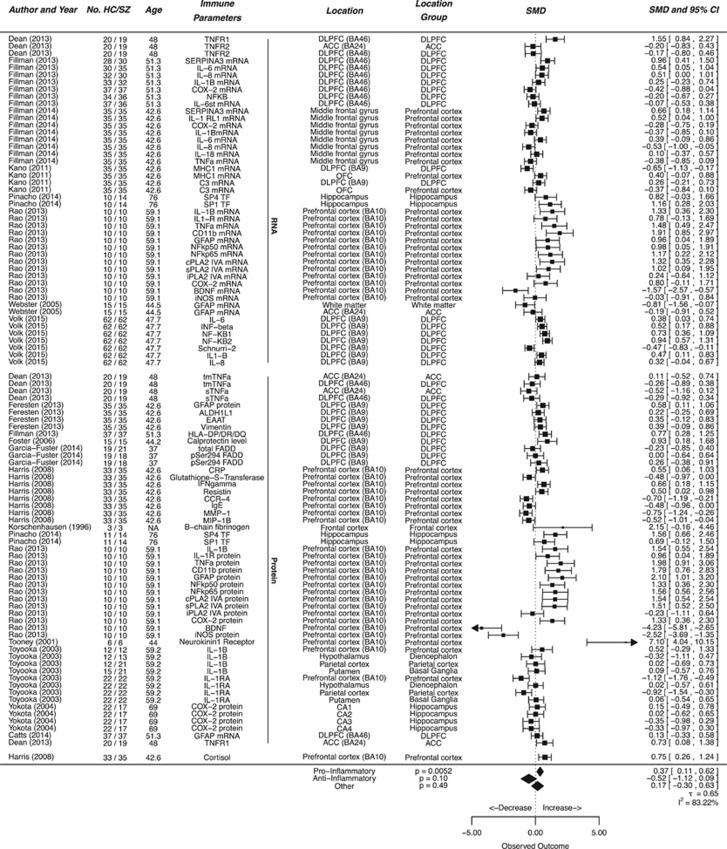
Molecular parameters in postmortem brains of schizophrenia patients. Forest plot showing molecular parameters measured in the brains of schizophrenic patients compared to controls. Values are expressed as standardized mean differences (SMDs) with 95% confidence intervals (CI). The column ‘No. HC/SZ' denotes the number of patients in the healthy control and schizophrenic groups. The column ‘Immune Parameter' denotes the parameter measured. The plot is divided in subsections showing study cohorts in which RNA expression, protein expression and hormone levels are measured. The column ‘Location' indicates the brain area under investigation as reported by the authors of the individual study. The diamonds at the bottom indicate the pooled estimate obtained by multilevel random effects meta-analysis.

We sought to further strengthen our findings by additionally reviewing alterations in gene expression in the brains of schizophrenic patients on transcript and protein level. The included studies investigated a great number of parameters, which complicated interpretation of overall analyses. We therefore divided the markers in this category into pro-, anti-inflammatory or other according to their association with increased activity of the immune system (Figure 3). The ‘other category' contained structural proteins such as vimentin or macroglial markers such as glia fibrillary acidic protein (GFAP).

We identified 14 studies that investigated expression of proinflammatory genes, including a total of 330 patients and 323 controls. The group of anti-inflammatory molecular markers comprised 3 studies, on 94 patients and 94 controls. A significant increase was observed in the overall expression of proinflammatory molecular components (effect size=0.37 SMDs (95% CI 0.11–0.62), *P*=0.0052). Sub-analyses on RNA versus protein expression in proinflammatory markers did not reveal any significant difference (*P*_RNA vs Prot_=0.39, effect size for protein=0.33, 95% CI 0.01–0.65, for RNA 0.39, 95% CI −0.06 to 0.72). No significant expression in anti-inflammatory markers was found between patients with schizophrenia and healthy controls (effect size is −0.52 SMDs, (95% CI 0.09 to −1.12, *P*=0.10). We further attempted a subgroup-analysis on markers that were assessed in more than three separate studies. In this analysis however no consistent effects were found. (Supplementary Figure 1)

### Sensitivity analyses

Sensitivity analyses were performed in order to assess the robustness of the observed findings. A funnel-plot of the studies investigating alterations in microglia numbers shows a pronounced right-skewed result (Supplementary Figure 2). This indicates that the observed increase in microglial numbers in schizophrenia patients, is substantially dependent on a few study cohorts rather than a stable and generalized effect. No irregularities were observed in macroglia numbers or expression of proinflammatory molecules (Supplementary Figures 3 and 4).

### Effect moderators

**Figure 4:**
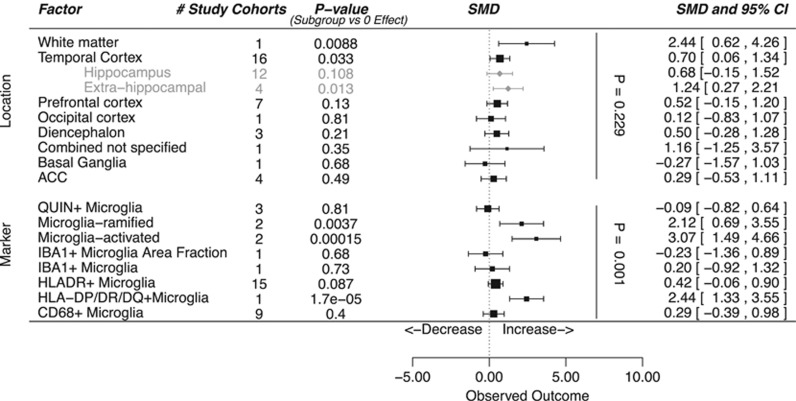
Meta-regression in studies investigating microglia. Forest plot showing location and markers as effect moderators on differences in cellular parameters. ‘*P*-value (Subgroup vs 0 effect) indicates the amount of evidence for alterations in a given brain region or effects observed using a given marker. The vertical *P*-values indicate the added value of the moderators location and cell type in the meta-regression model. ACC, anterior cingulate cortex; SMD, standardized mean difference.

**Figure 5:**
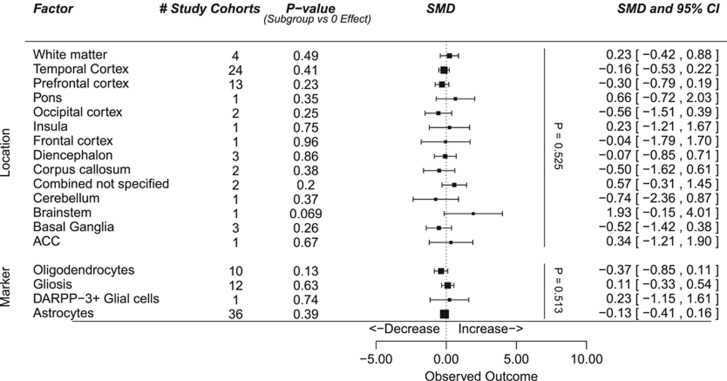
Meta-regression in studies investigating macroglia. Forest plot showing brain region and markers as effect moderators on differences in cellular parameters. ‘*P*-value (Subgroup vs 0 Effect)' indicates the amount of evidence for alterations in a given brain region or effects observed using a given marker. The vertical *P*-values indicate the added value of the moderators brain region and cell-marker in the meta-regression model. ACC, anterior cingulate cortex; SMD, standardized mean difference.

We further performed meta-regression analysis in the subset of studies investigating microglia to identify potential sources of heterogeneity between the studies. As there was little consistency in the molecular markers measured in the included studies, we did not perform meta-regression on this category. The inclusion of brain region as a factor in a meta-regression model could not explain a significant proportion of the variance (*P*=0.23 for the difference between brain regions). Among regions the area with most evidence for alteration in microglia presence was the temporal cortex with a significant increase in cell density (*P*=0.033, from 16 study cohorts). Further subdivision indicates that the effects were mainly found in brain areas outside the hippocampus, Brodmann's areas 20 and 22. One group studied white matter and observed a significant increase of microglia density (*P*=0.0088; Figure 4). Substantial differences in study results were found depending on methodological aspects and the different markers used to identify microglia in brain tissue sections (*P*=0.0003). In the category of macroglia no significant differences between schizophrenic patients and controls were found, irrespective of brain area or cell type (see Figure 5).

Not all of the studies summarized here reported on the gender of the included patients; data were available on 453 males (64.1%) and 254 females (36.0%). This represents a male:female ratio of 1.78:1.0. In addition we calculated the prevalence of deaths caused by suicide in studies reporting on cause of death. In our sample for 98 patients suicide was the cause of death compared to 426 patients with a natural cause of death, indicating a prevalence of 18.7%. Meta-regression on age at death, suicide as cause of death, pH-value of the investigated specimen, duration of illness, and gender revealed that these effect moderators did not significantly affect our results (see Supplementary Figures 5, assessment performed with microglia numbers).

## Discussion

Results of postmortem studies so far have been inconclusive as to whether there is a net effect of immune activation of the CNS in schizophrenia and whether such an effect is either on the cellular or the molecular level or both. A recent systematic review by Trepanier *et al.*^30^ has pointed out evidence for neuroinflammation in postmortem brains of schizophrenia patients. In this study, we investigated by quantative review the involvement of different components of the immune system in postmortem brain studies of patients with schizophrenia-spectrum disorders.

On the cellular level we found a significant increase in microglia density in patients with schizophrenia as compared with matched controls. Sensitivity analyses showed an inhomogeneous effect, possibly dependent on the method used to identify microglia or the brain region investigated. The number or activity state of macroglia did not differ between patients and controls. Further investigation showed an overall upregulation in the expression of proinflammatory genes in patients with schizophrenia.

### Cellular components

An important finding was the increased density of microglia in the patient group. In healthy tissue, microglia together with perivascular macrophages are the main myeloid cell populations of the brain parenchyma. During states of brain disease, monocytes may infiltrate the CNS and acquire a macrophage phenotype. These cells are referred to as the ‘infiltrating' macrophages. Microglia are myeloid cells that originate from erythromyeloid progenitors that have entered the CNS early during embryonic development. This myeloid compartment is maintained independent from the bone marrow.^31^ Although microglia and macrophages have overlapping functions and largely express similar markers, the cells have a different ontogeny and renewal mechanisms, suggesting that these populations have different functions in pathological states.^32, 33^ The most commonly used markers to identify microglia, such as HLA-DR and CD68, do not clearly distinguish between the different myeloid cells populations,^32, 34, 35^ in particular microglia and macrophages. Although under general discussion within the field,^36^ the blood–brain barrier is often hypothesized to be intact in patients with schizophrenia.^37^ Therefore, the most likely explanation of increased number of myeloid cells demonstrated in this study is an increase in the number of microglia resulting from local expansion in the CNS.

In our meta-analysis we chose to keep the nomenclature of the cells as presented in the original studies, categorizing them as microglia but allowing for the possible involvement of other myeloid cells such as macrophages.

Further studies using recently identified markers^35^ specific for macrophages or microglia, would shed light on the exact origin of the increased number of myeloid cells observed in this study. In the resting brain, myeloid cells are involved in homeostatic processes but also scan the surrounding tissue for injury. Upon detection of injury signals, the cells adopt an activated phenotype. The only study in our meta-analysis that specified the density of resting (ramified) and activated microglia,^38^ found that the number of microglia in both states were increased, but activated microglia even more so. This is in line with the conclusions of three imaging studies using PET^12, 13, 14^ where a significantly larger binding potential to the tracer was observed indicating an increased density of (possibly activated) microglia cells. Different PET tracers can be used to visualize the presence of microglia, for instance the PK11195 tracer, a ligand for the benzodiazepine receptor. Two independent studies observed increased binding of this tracer in schizophrenia, which was most pronounced in the temporal lobes.^12, 13^ A recent study on the same PET-tracer could not replicate this finding, however.^39^ Three further studies using different tracers, Kenk *et al.*^40^ and Hafizi *et al.*^41^ using the [^18^F]-FEPPA tracer and Coughlin *et al.*^42^ using the [11C]DPA-713 tracer similarly did not find significant differences between patients and controls. Another recent PET study, however, using a similar tracer, PBR 28, observed higher microglial activity both in individuals with subclinical symptoms at ultra-high risk for psychosis.^14^

Interestingly, a recent review on the effects of stress on microglia shows that both psychosocial stressors early in life as in adulthood can cause elevated microglial activity predominantly in hippocampal regions.^43^ As (early life) stress has been associated with elevated proinflammatory cytokines in childhood and an increased risk of developing psychotic disorders in young adulthood,^44^ stress may have a role in the increased microglia density and activity observed in schizophrenia patients.

Furthermore, closer inspection of affected brain regions in these PET imaging studies showed a most pronounced increase in activity in the temporal cortex.

In the present study, we observed similar results in meta-regression on brain region. In addition, a recent large meta-analysis on brain magnetic resonance imaging found significant subcortical brain abnormalities in schizophrenia patients versus controls, of which the decrease of hippocampal regions was most pronounced.^45^ The temporal cortex is known to be involved in higher order processing on sensory, emotional and cognitive level.^46^ Impairment of temporal lobe functioning such as observed in epilepsy and encephalitis, often manifest as a psychotic disorder. A predilection for inflammation in temporal cortex could therefore be a contributing factor in schizophrenia.^46^

When analyzing white matter, of which only one group specifically studied the involvement of cellular immune components, a significantly increased microglia density was found; indicating that increased immune activation could be present in both gray and white matter. This is in line with Pasternak *et al.*^47^ whose research showed increased free water in the white matter tracts of schizophrenia patients, which may be associated with an increased inflammatory status.

In contrast to microglia density, the density of macroglial cells appears not to be altered in schizophrenia compared with controls. A parallel increase in the number of astrocytes together with microglia could be expected in an acute inflammatory state,^48^ but this appears not to be the case in patients with schizophrenia.^49^ In chronic reactive gliosis, astrocytes do not proliferate but become hypertrophic.^16^ One of the studies in our sample also looked at astrocyte morphology and found a more prevalent occurrence of hypertrophic astrocyte morphology in individuals with schizophrenia who also had increased expression of inflammatory markers.^50^

### Molecular components

In an attempt to further substantiate the role of components of the immune system in the etiology of schizophrenia, we also summarized studies examining changes in gene and protein expression in schizophrenia patients. As all included studies had different primary hypotheses, a multitude of different parameters was assessed. In order to obtain a rough direction of effect, we divided the parameters into groups based on their association with increased or decreased activity of the immune system. Despite the substantial heterogeneity we observed a significant trend towards increased expression of proinflammatory genes in the brains of patients with schizophrenia. We created a summary estimate by using SMDs, a standardized effect measure that is independent of natural units. The interpretation of the summary is difficult, however, and should only be viewed as a rough measure of effect direction and size. We intended the inclusion of gene expression primarily as confirmation of the histological alterations in cellular composition. Overall, we observed a significant effect towards increased expression of proinflammatory genes. This finding supports our hypothesis regarding the involvement and activation of the immune system in the etiology of schizophrenia.

Microglia in activated state are known to produce proinflammatory cytokines (interleukin (IL)-1β, IL-6 and tumor necrosis factor (TNF)-α) while in resting state produce neurotrophic factors, such as brain-derived neurotrophic factor.^51^ Among the proinflammatory cytokines measured in postmortem tissue, IL-1β, was most consistently altered, showing a substantial increase in four studies.^52, 53, 54, 55, 56^ Studies on different modalities such as peripheral blood and CSF, but also genetic studies, have consistently reported an increase of IL-1β in schizophrenia.^57, 58, 59^ Moreover, other proinflammatory such as IL-6 and TNFα molecules were also shown to be increased in peripheral blood of patients with schizophrenia.^60^ The difference in anti-inflammatory gene expression did not become significant although there appeared to be a trend towards lower expression in patients, which may be caused by low power, as only three studies could be included. More research is needed to further clarify these findings.

We were not able to confirm our secondary finding regarding differential involvement of brain regions as observed in microglia. Most of the included studies focused on the prefrontal cortex and the studies involving the temporal cortex solely investigated the hippocampus.

### Effect moderators

Increased volume of the lateral ventricles and a general loss in brain volume are the most consistently observed brain alterations in schizophrenia.^61^ Their underlying pathophysiology, however, remains unclear. As increased activation of the CNS immune system leads to decreased production of brain-derived neurotrophic factor and other neurotrophic factors, this process, together with the production of neurotoxic proinflammatory factors such as IL-6, TNFα and IL-1β, may contribute to brain volume loss in schizophrenia. Immune activation is often expected to be most pronounced in the early stages of the disease when pronounced brain volume loss occurs.^62^ Postmortem studies, which mainly describe the late stages of schizophrenia, could therefore be considered not representative for the status of the CNS immune system in patients with a first psychotic episode. However, the advanced age of our postmortem sample combined with our findings of increased proinflammatory markers could challenge this view. Whether the association of these changes in immunes processes and schizophrenia is an aspect of the clinical state (for example, acute psychotic symptoms) of the disease remains to be defined. Only one group in our sample, conducting two studies, have subdivided their total population in a low- and high inflammatory group defined by high expression of proinflammatory cytokines such as IL-1b, IL-6, IL-8 and high SERPINA3 mRNA expression. The first study showing high inflammatory group primarily consisted of schizophrenia patients as opposed to controls, also with significantly higher mRNA expression of proinflammatory cytokines than the remaining schizophrenia patients, suggesting a specific subset.^53^ In the second study this was replicated, with findings of concomitant alterations in stress signaling, for which also a trend was seen in a group of patients with a bipolar disorder.^54^ Further research to target a specified group of schizophrenia patients most affected by neuroinflammation is needed.

The effect of duration of illness on the presence of immune markers in the brain could further provide direction in this matter. In our analysis, however, the effect of duration of illness was not revealed as a significant factor. So far, only one postmortem microarray study analyzed brain tissue of patients with long and short duration of illness^63^ and, surprisingly, reported strongest indications of increased inflammation in the later stages of the illness. This could point to a more general status of CNS immune activation, which shows gradual increase with older age.^64^

Our sample has some distinct features in the distribution of gender and the prevalence of suicide. Although the distribution of gender in our sample is in favor of men with a ratio of 1.78, no significant differences between sexes were found. Although suicide as a cause of death was not found to be a significant covariate, the prevalence of suicides in our included sample of 18.7% was quite substantial. One of the groups included in our sample, Steiner *et al.*,^65^ link their finding of higher microglia densities in suicide, to a state of pre-suicidal stress in which microglia are more activated and produce proinflammatory cytokines. However, this finding has so far not been replicated in other studies. More research is needed to define the impact of suicidal behavior on specific immune components of the brain or vice versa.

Another moderator we assessed was the acidity of the tissue. Decreased pH-levels have previously been linked to increased brain temperature and an increased permeability of the blood–brain barrier, similar to the inflammatory response after focal trauma;^66^ this could suggest that pH-decrease is a marker of inflammation or tissue damage in the brain. Moreover, Olah *et al.*^67^ showed that microglia are very sensitive to the acid level of postmortem brain material, in pH values ranging from 6.2 to 6.7 only in one out of five samples a reasonable microglia population was found. In the included studies, the pH of the material was rather low (mean value 6.4), which could indicate that there is an underestimation of the actual myeloid cell density in the included subjects due to high acidity in different postmortem samples.

### Strengths and limitations

We believe this is the first study to summarize and quantitatively review inflammation in postmortem brain tissue in relation to schizophrenia. When interpreting the results of this study, it should be borne in mind that there was substantial heterogeneity among the studies in the analyses with inter-study heterogeneity >50 % of the variance in both the cellular and molecular category. This heterogeneity is inherent to pre-clinical meta-analyses, as most of the included studies have subtly differing research questions, and accompanying different designs. The strengths of the pooled estimates provided in this meta-analysis are inversely proportional to the diversity of the pooled parameters. Also, the different cellular markers stain for largely overlapping cell populations within the main cellular categories of microglia, astrocytes and oligodendrocytes. In the molecular studies TNFα and IL-1β for instance, although being different cytokines, are part of the same inflammatory cascade, however, and expression is likely to be correlated: an increase in one parameter will be accompanied by a similar relative increase in the other. As our study only examines relative increases via SMDs, pooled estimates are valid if correlation between parameters is high. Studies in peripheral blood plasma show that proinflammatory cytokines are usually highly correlated (*R*~0.6–0.7)^68, 69, 70^ It is unknown how the different pro- and anti-inflammatory makers assessed in the included studies are (cor)related in the brain however, and the pooled estimates should be therefore be interpreted with utmost caution.

Another limitation of meta-analysis is that analyses and evidence are restricted to the original study designs. Some parameters have been extensively investigated in multiple studies, whereas other promising candidates have been assayed in only a single study cohort.

As an overview was given on all studies from 1970 until the present we see a difference in techniques used over time. Older studies mainly used hypothesis-directed assays such as western blotting, fluorescent *in situ* hybridization and reverse transcription PCR (RT-PCR), whereas in recent studies also hypothesis generating multiplex assays such as micro-arrays are used. Whereas meta-analysis of microarray experiments is possible, it must follow the same explorative and unbiased design as the original studies, in which thousands of hypotheses are tested simultaneously and a false discovery rate-limiting correction is applied *post hoc.* As these studies are epistemologically different from conventional hypothesis-directed research, we have chosen to exclude them from this meta-analysis, but recommend that this be a focus of future research.

Although we investigated the effect of several moderators, the effect of antipsychotic medication could not be studied. Both typical and atypical antipsychotics have anti-inflammatory properties and decrease cytokines such as IL-1β, IL-6 and TNF- α.^71, 72, 73, 74^ This likely affects our findings, and especially can contribute to the variation found in the molecular category. Another confounding factor, potentially enhancing proinflammatory processes is smoking. The majority of patients with schizophrenia (70–85%) are smokers,^75^ three times more than the general population.^76^ In our study, only one group^77^ specified results for smokers and non-smokers, finding a reduction of MHC class I protein expression in the dorsolateral prefrontal cortex in non-smoking schizophrenia patients, not seen in smoking schizophrenia patients. Finally, an estimated 33% of schizophrenia patients suffer from metabolic syndrome,^78^ again making the patient population more prone for increased levels of CNS inflammation.^79^ At this point, we are unable to disentangle the effects of medication use, smoking and metabolic syndrome on the observed differences in CNS immune status.

The finding of an increase in microglial cell density together with increased proinflammatory gene expression further substantiates the hypothesis of neuroinflammation as a component in the pathogenesis of schizophrenia. More research is needed to specifically define whether the activated immune system in schizophrenia must be seen as a cause or a consequence of the disease. In both scenarios, these results are highly relevant; an immune involvement in schizophrenia could aid the search for new treatment options in schizophrenia. For instance, by pointing out an important pioneering field of research into the efficacy of drugs with anti-inflammatory capacities (for example, NSAIDs, statins, estrogens or even corticosteroids), lifestyle adaptations (that is, reducing stress, more exercise and different sleep patterns), food supplements (*n*-acetylcysteine or omega-3 fatty acids), probiotics or other preventive or therapeutic interventions for patients with schizophrenia. It is currently unknown whether such interventions could be targeted to a specific subgroup of patients with high inflammatory stigma, and whether this selection could be based on peripheral or central immune measurements.^80, 81^

In conclusion, we found a significant increase in microglia and proinflammatory gene expression in the postmortem brains of schizophrenia patients as compared with controls. It is likely that this effect is more pronounced in certain areas of the brain, such as the temporal cortex. We observed significant heterogeneity between study results due to differences in methodology and marker use. Systematic comparisons of different brain regions using novel markers and validated histological methods should be conducted to confirm the findings of this study. However, findings of this meta-analysis strengthen the hypothesis that neuroinflammation has a role in the pathogenesis of schizophrenia, which may give rise to much needed new prevention and treatment strategies.^82, 83, 84^
