# Supplementary material for: Immune involvement in the pathogenesis of schizophrenia: a meta-analysis on postmortem brain studies
Source: Transl Psychiatry. 2017 Mar 28;7(3):e1075–. doi: 10.1038/tp.2017.4 (PMC5404615; doi:10.1038/tp.2017.4)
Supplement: Supplementary Figures [file tp20174x1.pdf]

# Supplementary Material

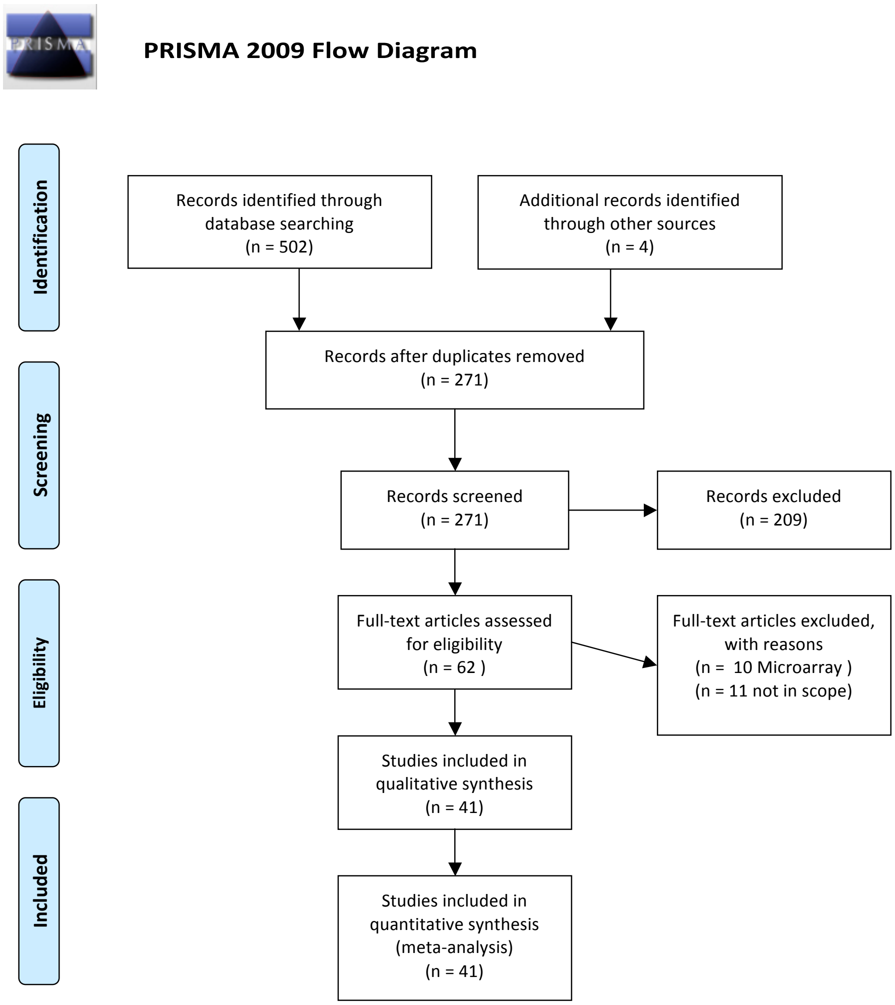


*From:* Moher D, Liberati A, Tetzlaff J, Altman DG, The PRISMA Group (2009). *P*referred *R*eporting *I*tems for *S*ystematic Reviews and *Meta-A*nalyses: The PRISMA Statement. PLoS Med 6(7): e1000097. doi:10.1371/journal.pmed1000097

**For more information, visit www.prisma-statement.org.**

Figure 1: PRISMA Diagram

**Supplementary Table 1: Classification of measured parameters in included studies:** Categories and Subcategory as used as classifiers in the manuscript are indicated, along with the originally quantified variable.

| Category | Subcategory | Measurement | Frequency |
|---|---|---|---|
| Cell | Lymphocytes | CD20+ Cells | 1 |
| Cell | Lymphocytes | CD3+ Cells | 1 |
| Cell | Macroglia | Astrocytes | 6 |
| Cell | Macroglia | Astroglial Density | 3 |
| Cell | Macroglia | Ependymal discontinuities | 1 |
| Cell | Macroglia | GFAP+ Cells | 25 |
| Cell | Macroglia | GFAParea fraction | 2 |
| Cell | Macroglia | Glial cells | 5 |
| Cell | Macroglia | Gliosis | 3 |
| Cell | Macroglia | Nodular gliosis | 1 |
| Cell | Macroglia | Olig. Nuclear Area | 1 |
| Cell | Macroglia | Olig. Nuclear Diam. | 1 |
| Cell | Macroglia | Oligodendrocytes | 6 |
| Cell | Macroglia | S100B+ Glia | 2 |
| Cell | Macroglia | Subventricular rosettes | 1 |
| Cell | Microglia | CD68+ Microglia | 9 |
| Cell | Microglia | HLA-DP/DR/DQ | 1 |
| Cell | Microglia | HLADR+ Microglia | 8 |
| Cell | Microglia | IBA1+ Cells | 1 |
| Cell | Microglia | Microglia | 6 |
| Cell | Microglia | Microglia-activated | 2 |
| Cell | Microglia | Microglia-ramified | 2 |
| Cell | Microglia | Microglial Density | 3 |
| Cell | Microglia | QUIN+ Microglia | 3 |
| Molecular | Hormone | Cortisol | 1 |
| Molecular | Protein anti-inflamm | BDNF | 1 |
| Molecular | Protein anti-inflamm | IL-1R protein | 1 |
| Molecular | Protein anti-inflamm | IL-1RA | 5 |
| Molecular | Protein other | ALDH1L1 | 1 |
| Molecular | Protein other | EAAT | 1 |
| Molecular | Protein other | GFAP protein | 2 |
| Molecular | Protein other | IgE | 1 |
| Molecular | Protein other | Vimentin | 1 |
| Molecular | Protein proinflamm | B-chain fibrinogen | 1 |
| Molecular | Protein proinflamm | CCR-4 | 1 |
| Molecular | Protein proinflamm | CD11b protein | 1 |
| Molecular | Protein proinflamm | COX-2 protein | 5 |
| Molecular | Protein proinflamm | CRP | 1 |
| Molecular | Protein proinflamm | Calprotectin level | 1 |
| Molecular | Protein proinflamm | Glutathione-S-Transferase | 1 |
| Molecular | Protein proinflamm | IFNgamma | 1 |
| Molecular | Protein proinflamm | IL-1B | 5 |
| Molecular | Protein proinflamm | MIP-1B | 1 |
| Molecular | Protein proinflamm | MMP-1 | 1 |
| Molecular | Protein proinflamm | NFkp50 protein | 1 |
| Molecular | Protein proinflamm | NFkp65 protein | 1 |
| Molecular | Protein proinflamm | Neurokinin1 Receptor | 1 |
| Molecular | Protein proinflamm | Resistin | 1 |
| Molecular | Protein proinflamm | SP1 TF | 1 |
| Molecular | Protein proinflamm | SP4 TF | 1 |
| Molecular | Protein proinflamm | TNFa protein | 1 |

| Category | Subcategory | Measurement | Frequency |
|---|---|---|---|
| Molecular | Protein proinflamm | cPLA2 IVA protein | 1 |
| Molecular | Protein proinflamm | iNOS protein | 1 |
| Molecular | Protein proinflamm | iPLA2 IVA protein | 1 |
| Molecular | Protein proinflamm | pSer294 FADD | 2 |
| Molecular | Protein proinflamm | sPLA2 IVA protein | 1 |
| Molecular | Protein proinflamm | sTNFa | 2 |
| Molecular | Protein proinflamm | tmTNFa | 2 |
| Molecular | Protein proinflamm | total FADD | 1 |
| Molecular | RNA anti-inflamm | BDNF mRNA | 1 |
| Molecular | RNA anti-inflamm | IL1-R mRNA | 1 |
| Molecular | RNA other | GFAP mRNA | 3 |
| Molecular | RNA other | MHC1 mRNA | 2 |
| Molecular | RNA proinflamm | C3 mRNA | 2 |
| Molecular | RNA proinflamm | CD11b mRNA | 1 |
| Molecular | RNA proinflamm | COX-2 mRNA | 3 |
| Molecular | RNA proinflamm | IL-1 RL1 mRNA | 1 |
| Molecular | RNA proinflamm | IL-18 mRNA | 1 |
| Molecular | RNA proinflamm | IL-1B mRNA | 2 |
| Molecular | RNA proinflamm | IL-1BmRNA | 1 |
| Molecular | RNA proinflamm | IL-6 mRNA | 2 |
| Molecular | RNA proinflamm | IL-6st mRNA | 1 |
| Molecular | RNA proinflamm | IL-8 mRNA | 2 |
| Molecular | RNA proinflamm | NFKB | 1 |
| Molecular | RNA proinflamm | NFkp50 mRNA | 1 |
| Molecular | RNA proinflamm | NFkp65 mRNA | 1 |
| Molecular | RNA proinflamm | SERPINA3 mRNA | 2 |
| Molecular | RNA proinflamm | SP1 TF | 1 |
| Molecular | RNA proinflamm | SP4 TF | 1 |
| Molecular | RNA proinflamm | TNFR1 | 2 |
| Molecular | RNA proinflamm | TNFR2 | 2 |
| Molecular | RNA proinflamm | TNFa mRNA | 2 |
| Molecular | RNA proinflamm | cPLA2 IVA mRNA | 1 |
| Molecular | RNA proinflamm | iNOS mRNA | 1 |
| Molecular | RNA proinflamm | iPLA2 IVA mRNA | 1 |
| Molecular | RNA proinflamm | sPLA2 IVA mRNA | 1 |

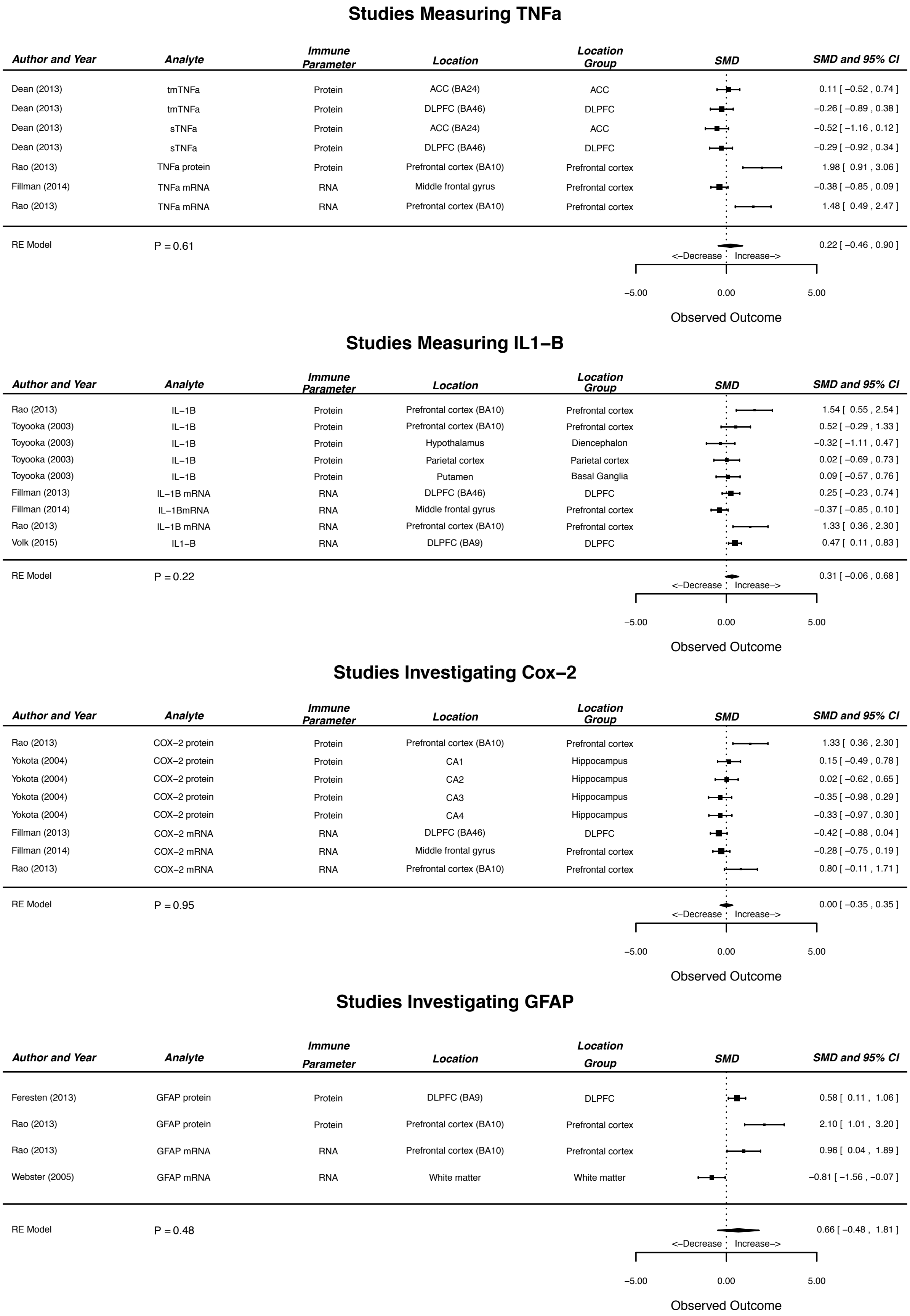


Figure 2: **Subgroup Analysis on Molecular Parameters:** Forest plots depicting substudies on frequently assessed molecular markers

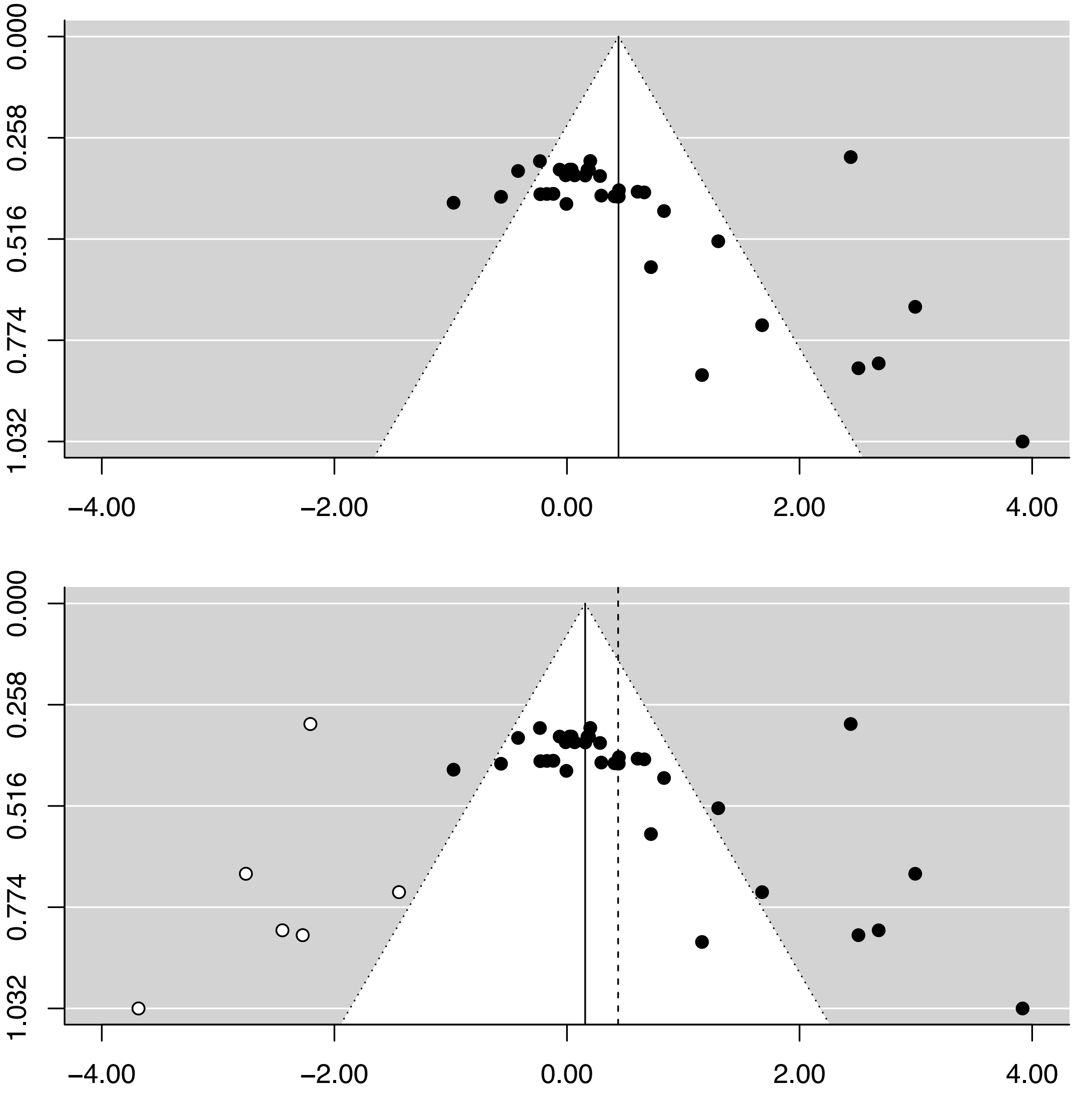


Figure 3: **Funnelplot of Studies Investigating Microglia.** This graphic shows the Standard Error of study cohorts on the y-axis and the cohorts' point estimates on the x-axis, individual studies are depicted as closed circles (top). It is expected that individual study estimates are symmetrically distributed around the pooled mean, with more precise studies (smaller SE, higher on the y-axis) being closer to the pooled mean (as indicated by the white triangle). The present graph shows an increased number of studies right of the pooled mean, indicating potential irregularities in inter-study consistency. A stabilized estimate by the 'trim and fill' method by Duval and Tweedie, in which 'missing' studies are imputed, shows that the increase in microglial number is highly dependent on the few outlying studies (p = 0.008). The bottom graph shows the imputed studies (open circles) and the shift of the summary estimate (dotted line without imputed studies, solid line with imputed studies).

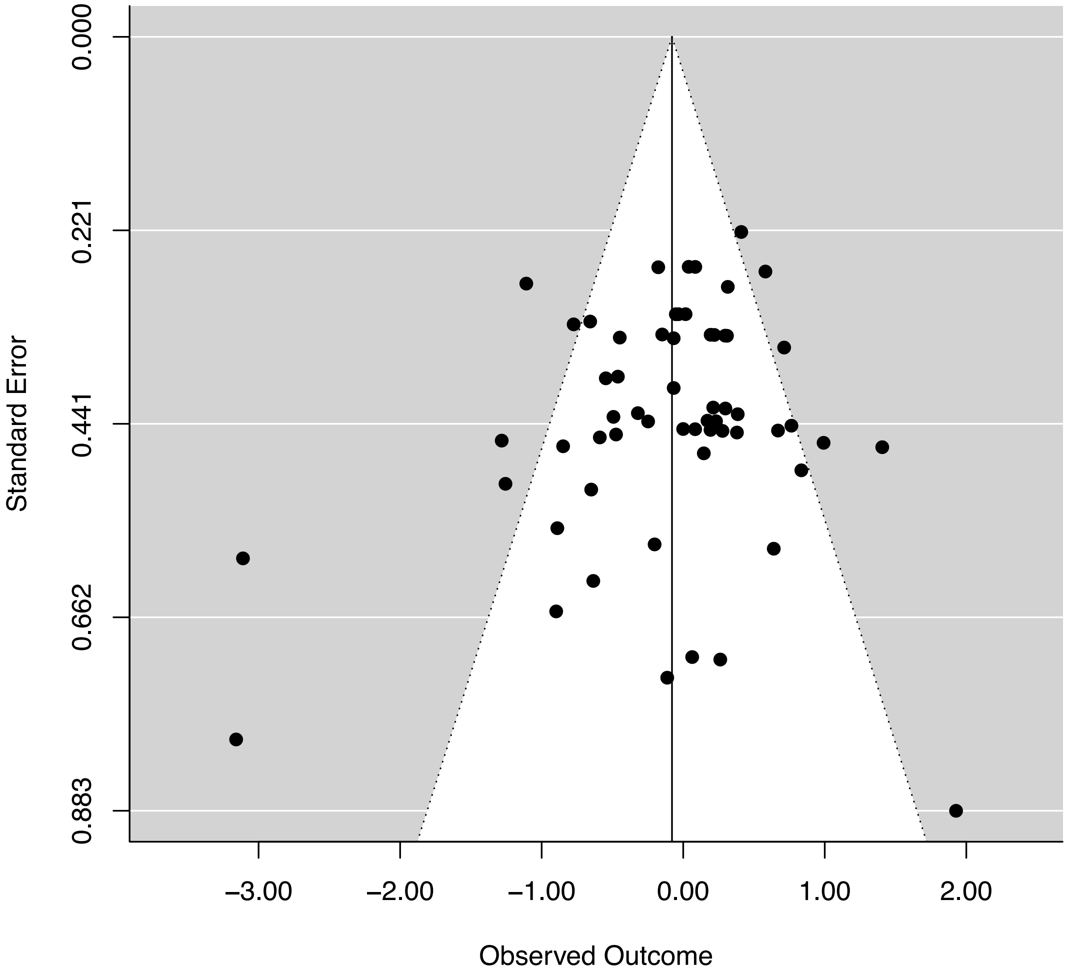


Figure 4: **Funnelplot of Studies Investigating Macroglia.** The present graph shows no assymmetry (Egger's regression test, p = 0.19)

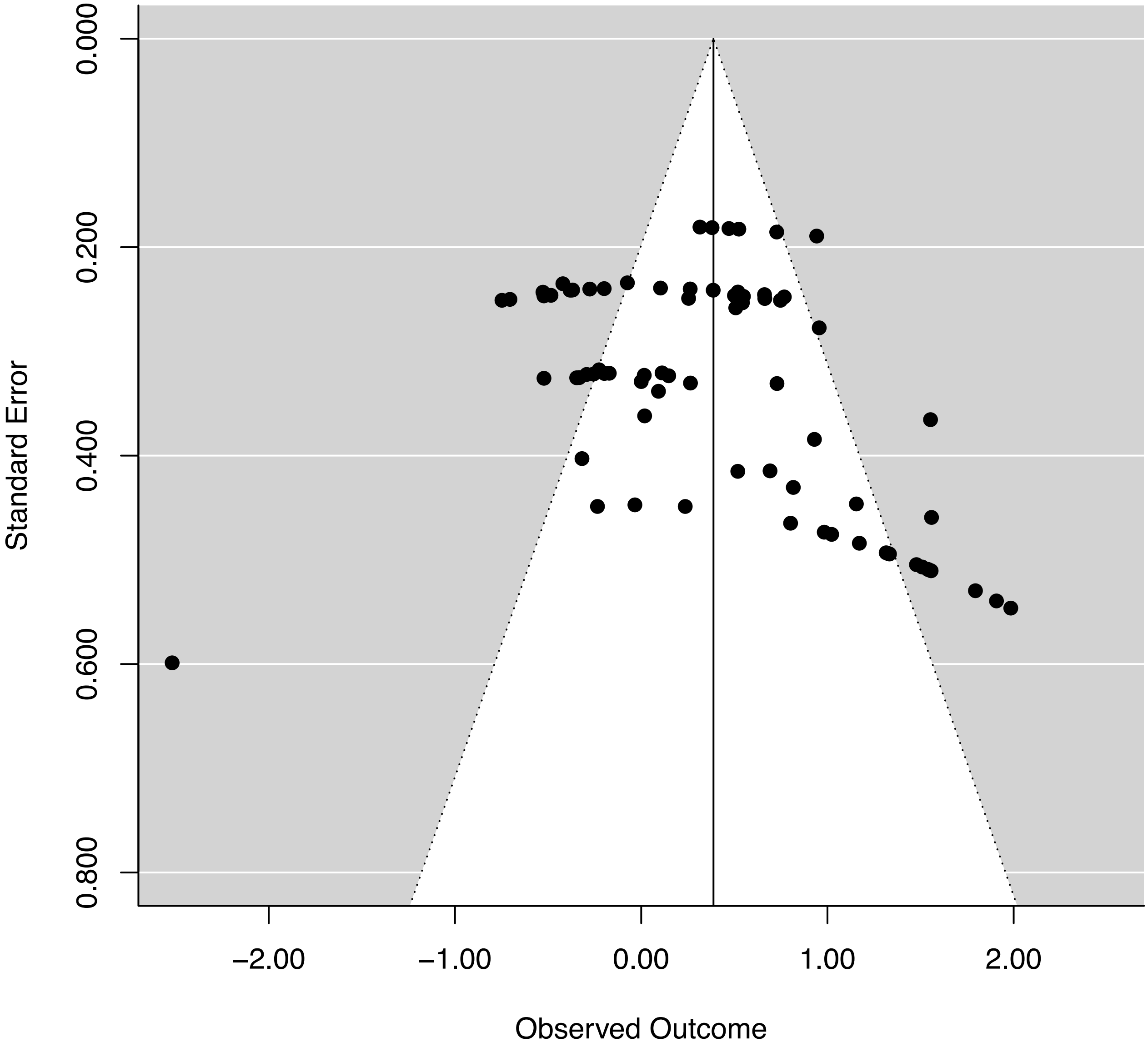


Figure 5: **Funnelplot of Studies Investigating Pro-Inflammatory Molecules.** The present graph shows no assymmetry (Egger's regression test, p = 0.50)

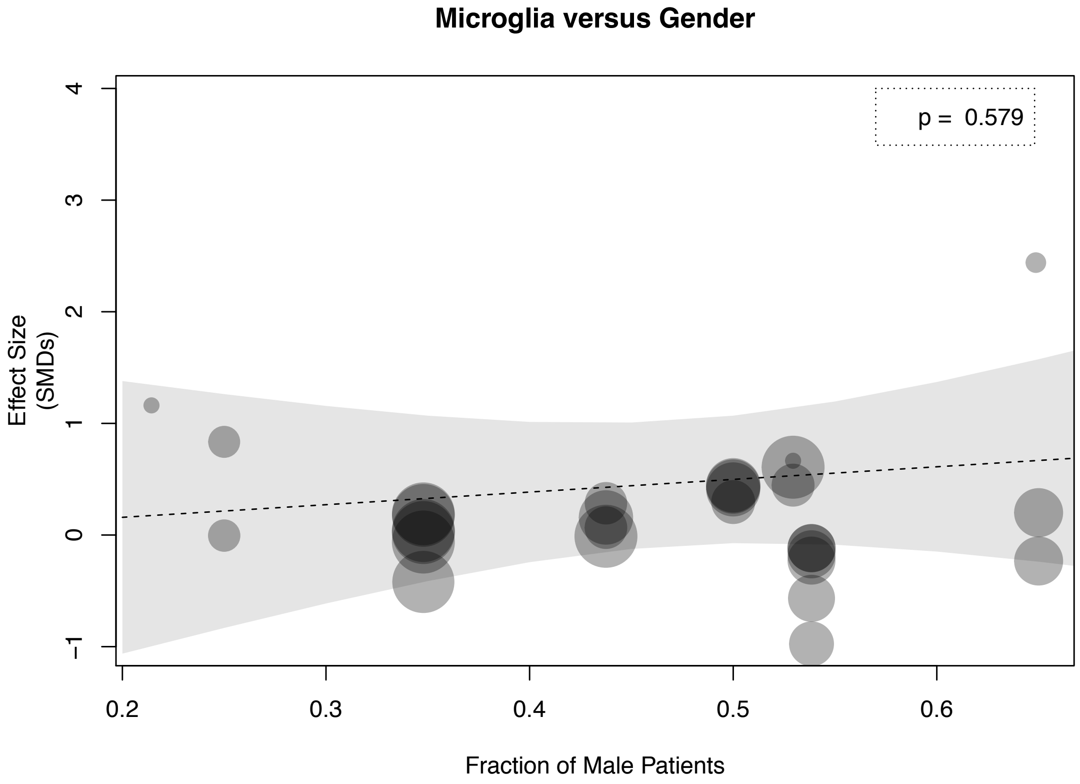


Figure 6: **Effect of Gender on Microglial Cell Increase:** Meta-analytic scatterplot showing the relationship between the fraction of male subjects in a given study cohort and the increase in microglia in patients versus controls. The size of each dot is proportional to the weight of each study cohort in the nested model. There was no significant relationship between proportion of male patients and increase in microglia (p=0.58)

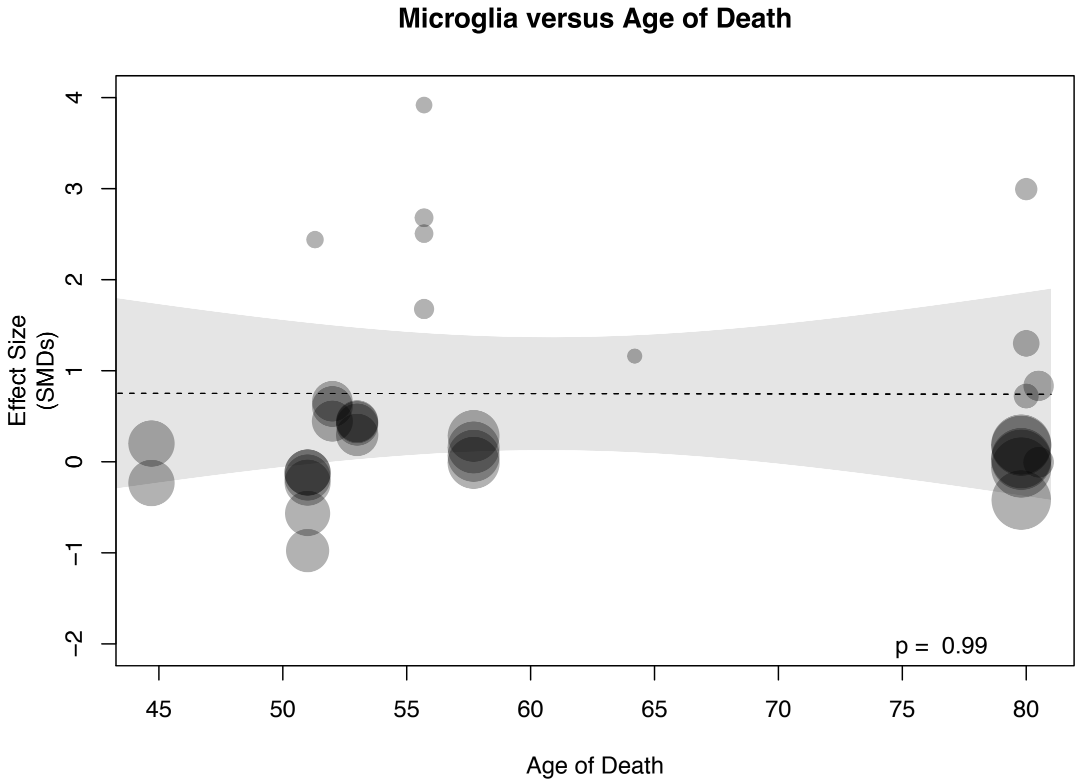


Figure 7: **Effect of Age of Death on Microglial Cell Increase:** Meta-analytic scatterplot showing the relationship between the average age of death in a given study cohort and and the increase in microglia in patients versus controls. The size of each dot is proportional to the weight of each study cohort in the nested model. There was no significant relationship between average age of death and increase in microglia (p=0.99)

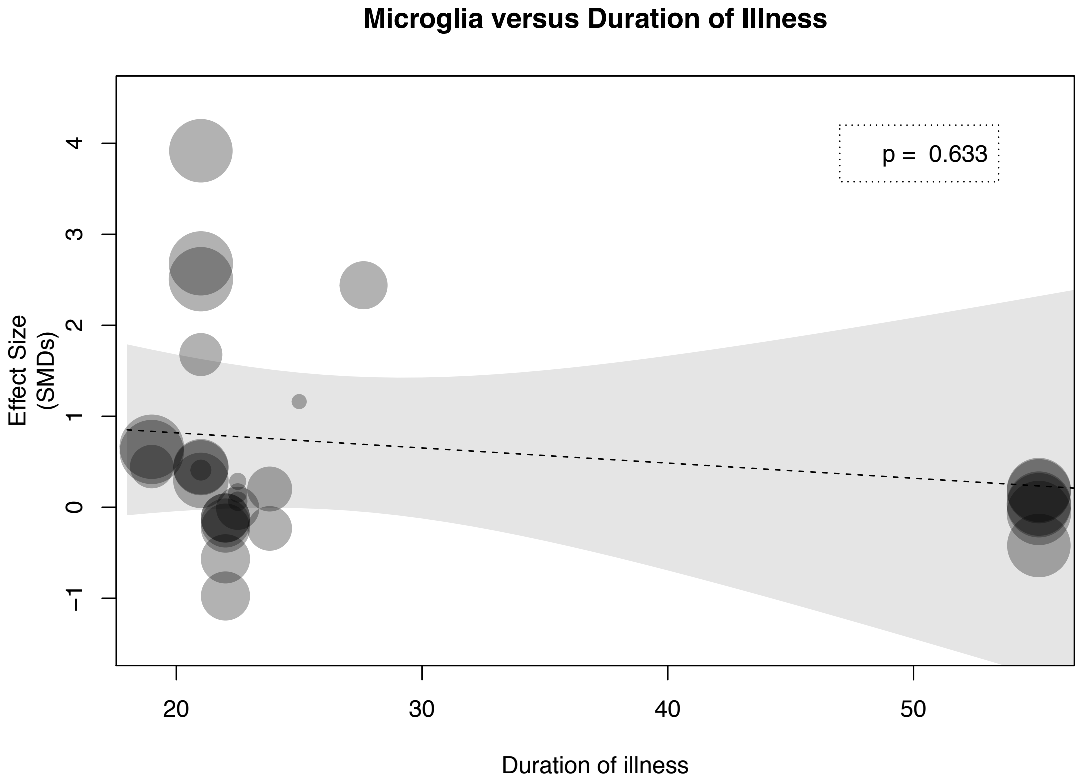


Figure 8: **Duration of Ilness and Microglial Cell Increase:** Meta-analytic scatterplot showing the relationship between the average duration of illness in schizophrenic patients in a given study cohort and and the increase in microglia in patients versus controls. The size of each dot is proportional to the weight of each study cohort in the nested model. There was no significant relationship between duration of illness and increase in microglia (p=0.63)

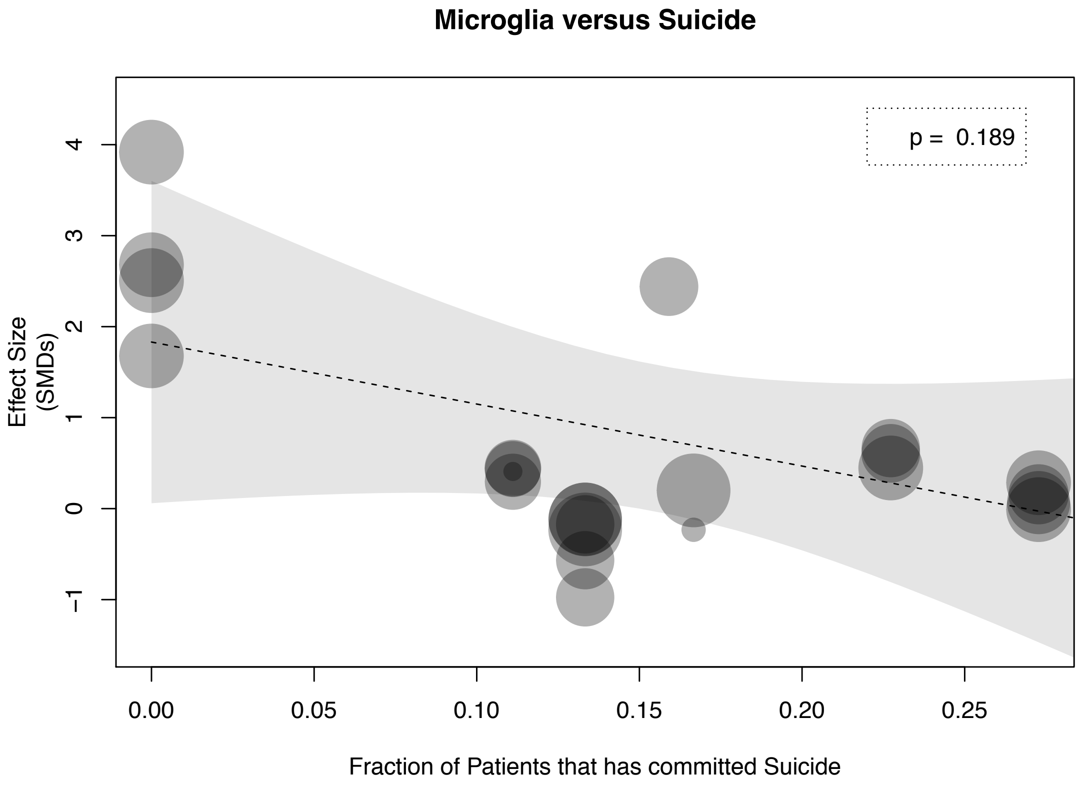


Figure 9: **Fraction of Patients that Commits Suicide and Microglial Cell Increase:** Meta-analytic scatterplot showing the relationship between proportion of death due to suicide in schizophrenic patients in a given study cohort and and the increase in microglia in patients versus controls. The size of each dot is proportional to the weight of each study cohort in the nested model, a single study can be represented by multiple dots if multiple microglia measurements have been performed. There was no significant relationship between proportion of suicides and increase in microglia (p=0.19).
